# Fusion of Unobtrusive Sensing Solutions for Sprained Ankle Rehabilitation Exercises Monitoring in Home Environments

**DOI:** 10.3390/s21227560

**Published:** 2021-11-13

**Authors:** Idongesit Ekerete, Matias Garcia-Constantino, Yohanca Diaz-Skeete, Chris Nugent, James McLaughlin

**Affiliations:** 1 School of Computing, Jordanstown Campus, Ulster University, Newtownabbey BT37 0QB, UK; m.garcia-constantino@ulster.ac.uk (M.G.-C.); cd.nugent@ulster.ac.uk (C.N.); 2 NetwellCASALA Advanced Research Centre, Dundalk Institute of Technology, A91 K584 Dundalk, Ireland; yohanca.diaz@dkit.ie; 3 School of Engineering (NIBEC), Jordanstown Campus, Ulster University, Newtownabbey BT37 0QB, UK; jad.mclaughlin@ulster.ac.uk

**Keywords:** unobtrusive sensing, data fusion, data mining, radar sensing, thermal sensing, sprained ankle, infrared thermopile array, home environment

## Abstract

The ability to monitor Sprained Ankle Rehabilitation Exercises (SPAREs) in home environments can help therapists ascertain if exercises have been performed as prescribed. Whilst wearable devices have been shown to provide advantages such as high accuracy and precision during monitoring activities, disadvantages such as limited battery life and users’ inability to remember to charge and wear the devices are often the challenges for their usage. In addition, video cameras, which are notable for high frame rates and granularity, are not privacy-friendly. Therefore, this paper proposes the use and fusion of privacy-friendly and Unobtrusive Sensing Solutions (USSs) for data collection and processing during SPAREs in home environments. The present work aims to monitor SPAREs such as dorsiflexion, plantarflexion, inversion, and eversion using radar and thermal sensors. The main contributions of this paper include (i) privacy-friendly monitoring of SPAREs in a home environment, (ii) fusion of SPAREs data from homogeneous and heterogeneous USSs, and (iii) analysis and comparison of results from single, homogeneous, and heterogeneous USSs. Experimental results indicated the advantages of using heterogeneous USSs and data fusion. Cluster-based analysis of data gleaned from the sensors indicated an average classification accuracy of 96.9% with Neural Network, AdaBoost, and Support Vector Machine, amongst others.

## 1. Introduction

Developments in sensing technologies have made positive impacts on many applications ranging from aerospace to automotive and healthcare. In the medical and health sectors, biosensors are deployed to monitor patient’s physiological conditions in hospital environments. Although the hospital environment has advantages such as direct supervision by specialists, appointment booking, logistical concerns, and a feeling of discomfort are some of the disadvantages.

Several activities and rehabilitation exercises can be monitored outside the hospital settings, such as assisted living and home environments [1]. Whilst the assisted living communities are noted for increased physical activities and socialisation opportunities, the home environment offers a more relaxed and convenient setting for rehabilitation exercises such as spinal cord injury rehabilitation exercises [2], cardio rehabilitation [3], post-stroke rehabilitation exercises [4,5], and Home-Based Ankle Rehabilitation [6].

Sprained ankles are injuries sustained due to inappropriate movement of the ankle. Although they are generally regarded as common injuries, they can degenerate into lifelong problems if not adequately treated. Common causes of a sprained ankle include ankle twists during a fall, awkward landing during sports activities, and stepping on uneven surfaces during walking or exercising. Sprained Ankle Rehabilitation Exercises (SPAREs) include a range of exercises aimed at helping recovery from these injuries [7]. Typical SPAREs include Range of Motion (RoM), balance, stretching, control, and strengthening exercises [8,9]. RoM exercises entail trying to move the ankle in all directions or some specific directions. The sufferer can take a sitting position, place the non-affected foot flat on the floor while attempting to move the affected foot in predetermined directions [8]. Common examples of RoM include controlled-resistant cycling and cord-assisted stretching. While RoM can be active, there is also evidence of the performance of passive RoM.

Balance and control exercises can involve the use of wobble boards with supports to avoid tipping [10]. It is often recommended for those with little or no pain on the sprained ankle. In addition, the duration of the exercise can span between a minute or more and up to six times in each session [8]. Balance exercises can include standing on the affected leg with the hands to the sides of the body or folded across the chest. Stretching exercises, on the other hand, involve extending the calf muscle and the Achilles tendon by pushing against a wall with the affected foot stretched out at the back. Strengthening exercises can include foot dorsiflexion, plantarflexion, inversion, and eversion [10]. Home-based SPAREs include a range of exercises that can be performed within the home environment to help recovery. Whilst some of these exercises can be performed using common devices such as elastic bands, wobble boards, and deformable plastic materials, sophisticated instruments, namely, treadmills, actuators, and cycling machines, can also be used.

The ability to monitor SPAREs can help to understand if the exercises and activities have been performed as prescribed. The monitoring devices can include a range of wearables and non-wearables, such as video cameras, gyroscopes, and accelerometers. Whilst video cameras pose issues ranging from privacy to storage capacity, accelerometers and gyroscopes require users to remember to charge and wear the devices. Battery life problems, data disruption, and a feeling of discomfort due to skin irritations from bands and cuffs are also common challenges with the use of wearables [11].

This study considers the sprained ankle strengthening exercises involving foot movement in the four fundamental directions. The present work aims to monitor SPAREs such as dorsiflexion, plantarflexion, inversion, and eversion using Unobtrusive Sensing Solutions (USSs) such as radar and thermal sensors. The main contributions of this paper include (i) privacy-friendly monitoring of SPAREs in a home environment, (ii) fusion of SPAREs data from homogeneous and heterogeneous USSs, and (iii) analysis and comparison of results from single, homogeneous, and heterogeneous USSs.

The remainder of the paper is organised as follows. Section 2 reviews Sensing Solutions (SSs) in home-based SPAREs monitoring, Section 3 presents the materials and methods for data acquisition and analysis, Section 4 presents experimental results, Section 5 discusses findings from the study, and Section 6 presents the conclusion.

## 2. Sensing Solutions in Home-Based Sprained Ankle Rehabilitation Exercises Monitoring

This section reviews the state-of-the-art in home-based SPAREs monitoring ranging from wearables to video-based Sensing Solutions.

### 2.1. Wearable Sensing Solutions

The use of wearables such as triaxial accelerometers, gyroscopes, and inertial sensors to monitor and estimate the orientations of a body has been widely researched [12,13,14,15,16]. The sensors’ usage has included measurement of the acceleration of the upper extremity post-stroke [17], indoor activity monitoring and classification [18,19,20,21,22,23], behaviour monitoring [24,25], and home-based motor functions rehabilitation [16], amongst others. In SPAREs, wearables such as foot-mounted inertial sensors have been used to estimate the orientation of the ankle [26]. Although inertial-based motion sensors can be used to obtain locomotion parameters, they are sometimes influenced by the temperature of the sensing environment [16,27].

Azizi et al. [7] proposed the use of a gyro-based system that included WiFi and microcontroller modules in determining ankle inclination and orientation. This study was noted to have provided significant improvement to the patients although it failed to estimate the percentage at which the participants recovered from the ailment. In [28], the use of a microcontroller-based system alongside an accelerometer to monitor the angular inclination of the ankle during SPAREs is proposed. The two degrees of freedom allowed by this system placed restrictions on the movement of the ankle during the exercises. The authors in [29] considered the major trends in SPAREs assistive and monitoring devices. Whilst devices with middle complexity were mostly wearables, high-complexity devices such as the Biodex multi-joint devices were noted for their bulkiness, high cost, professionalism requirements, and suitability for use in only clinical environments.

### 2.2. Video-Based Sensing Solutions

Video-based SS such as Kinect in home-based monitoring has become popular in recent years [30]. Kinect provides a 3D estimation of users’ postures and allows for real-time tracking of room occupants. It can also provide information on the joint segments of targeted persons [16,31,32]. Despite the remarkable usefulness of the Kinect sensors in games and rehabilitation, sensitivity to external infrared sources and reflective materials are the main drawbacks of their usage in home-based settings [33]. For these reasons, amongst others, such as privacy concerns [34], the Kinect sensor was not appropriate for this study [35].

A further study by [36] proposed a video game-based approach to SPAREs. The approach, which was measured against traditional exercises, was said to be effective in restoring the ankle functions based on indexes such as mood, pain perception, and readiness to return to active sports, amongst others. Furthermore, a game-based solution by [37] was compared with physical therapy and a controlled group. The study, which suggested six weeks of treatment, noted significant improvements in pain reduction. The authors of [38] proposed the use of augmented reality to help patients and physiotherapists in SPAREs. The study focused on the importance of using autonomous devices during SPAREs. In all the studies, monitoring processes were either performed using human observation or a video camera. While the former is prone to optical illusion and error, the latter requires higher storage space. In addition, video recordings can intrude into the privacy of the users of these technologies.

A privacy-friendly Sensing Solution (SS) was proposed by [39]. The work involved the use of a thermal imaging camera to study temperature variations in human ankles by comparing blood flow on sprained and non-sprained ankles. Although the thermal camera offered a privacy-friendly solution, parameters such as the range, velocity, and angle of the movement were not considered in the study. In addition, the extent of recovery was through human observation. Therefore, to ensure the effectiveness of these rehabilitation approaches and processes, Data Mining (DM) and Machine Learning (ML) algorithms should be used for their data analysis for pattern assessment and clustering. A comparison of the SSs is presented in Table 1.

Research evidence has suggested that most SPAREs monitored through face-to-face interaction and supervision of a physiotherapist can be monitored remotely [40,41]. Performing SPAREs in a remote environment, such as the home setting, can include following a set of instructional guides provided by a therapist [6,42,43]. The home-based approach can help address problems related to insufficient therapists and cost [44]. Unlike other rehabilitation exercises, monitoring SPAREs can be difficult owing to smaller angular movements of the ankle and the possibility of occlusion. Whilst video cameras pose privacy issues, Wearable Sensing Solutions (WeSSs) such as gyroscopes and accelerometers pose battery life and wearability problems. On the other hand, human observation can be prone to optical illusions and errors [11].

The present work proposes the use of unobtrusive and privacy-friendly SSs in the form of thermal and radar sensors to monitor SPAREs in a home environment. It also considers the use of DM and ML algorithms to perform Classification by Clustering (CbyC) of thermal images and blobs and other parameters such as range, speed, and the Angle of Approach or Retreat (AAR).

## 3. Materials and Methods

The methods employed in this work included data collection through experiments and preprocessing of the acquired (thermal and radar SSs) data. Others included thermal and radar sensors data processing using a Sensor Data Fusion (SDF) architecture. The sensors data were processed as single, homogeneous, and heterogeneous datasets. The SDF architecture presents two algorithmic pathways: Hierarchical Clustering Algorithm (HCA) and the K-Means++ Algorithm (KMA). Further analysis of the datasets with KMA is made to compare the averages of the model.

The statistical analysis method in the present work considered the averaging of model parameters against selecting the best model in order to aid subsequent computations. This includes obtaining the row averages of metrics such as Area Under the Curve (AUC), Classification Accuracy (CA), F1, Precision, and Recall for each model. Then, the row averages from KMA, HCA, fused homogeneous, and fused heterogeneous datasets are tabulated for column-wise averages. T-Test and descriptive analysis using ANOVA are performed on the column data referred to as KMA Averages (KMA-A), HCA Averages (HCA-A), data fusion averages from the side-facing and the front-facing ITA-32 sensors (SF-Fusion), and data fusion averages from the Infrared Thermopile Array (ITA-32) and Radar sensor (Rad-T Fusion). The averaging method was chosen against the best model method to avoid bias and present more data points in further analysis, such as T-Test and ANOVA.

**Figure 1:**
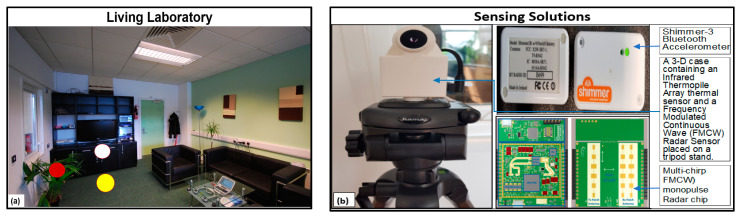
Sprained Ankle Rehabilitation Exercise setting: (**a**) the Living Lab where the study was conducted, and (**b**) the Sensing Solutions (SSs) used during the study. In Figure 1a, the red, white, and yellow spots indicted the locations of the side-facing SSs, the front-facing SSs, and the participants, respectively, during the study.

The experimental setup involved the use of multiple sensors to record SPAREs in a laboratory living room that mimics a real-world living room. They included (i) one Frequency Modulated Continuous Wave (FMCW) radar sensor, (ii) one Multi-Chirp Frequency Modulated Continuous Wave Mono-pulse (MC-FMCW-M) radar sensor, (iii) two ITA-32 sensors, and (iv) two Shimmer-3 accelerometer (S3BA) SSs. The S3BAs were used for ground truth measurement of velocity. While the radar and thermal sensors were mounted on tripod stands and placed for side and frontal views of the ankles, the two S3BAs were attached to the metatarsal to record the acceleration of each foot in the X, Y, and Z directions. The rationale for taking measurements from the front and side views was to avoid the effects of occlusion. The rationale for using multiple sensors was to allow for complementary monitoring, redundancy, and cross-validation of measurements. The setting of the study, including the Living Lab in which the study was conducted, the physical location of the participants, and the SSs are presented in Figure 1.

In Figure 1a, the red and the white spots indicate the location of the side-facing and the front-facing SS that were used to monitor the SPAREs. The yellow spot indicates where the participants sat during data collection. Whilst their legs were usually stretched towards the white spot (front-facing SS), side views of their actions were obtained with the side-facing SS to avoid occlusion. A total of 15 healthy participants, randomly selected from the School of Computing, took part in this study. In an upright sitting position, 20 directional movements were performed by the participants for 20 s on each leg. These included twisting the ankle in 4 fundamental directions of human ankle movement: (i) dorsiflexion, (ii) plantarflexion, (iii) eversion, and (iv) inversion. These movements were recorded simultaneously by all the sensors. The parameters measured included the angular orientation of the ankles and postures. Other parameters included their ranges and velocities at instances of dorsiflexion, plantarflexion, eversion, and inversion. However, data from the wearable sensors (S3BAs) were not considered in the data analysis. The rationale for not considering the S3BA data is that data analysis involving the S3BAs and the FMWC radar (aimed at comparing their velocity values) was considered in our previous study [45]. Data obtained by the thermal sensors were stored in a bespoke time-series database (*SensorCentral*) [46].

**Figure 2:**
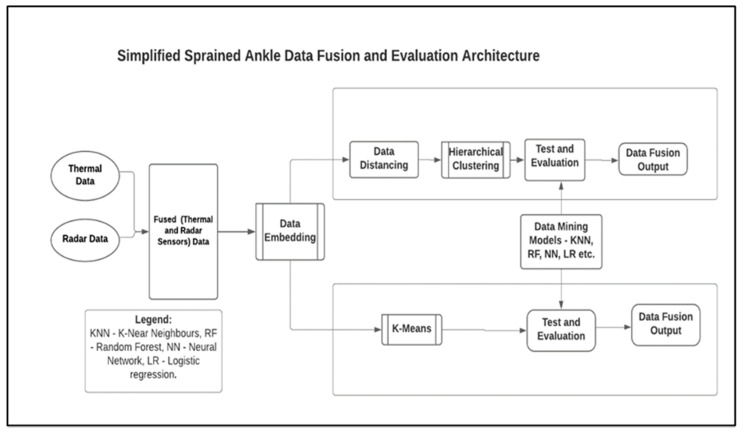
The Sensor Data Fusion and Evaluation Architecture.

Data collected during this study were analysed using an SDF architecture referred to as Modified Distributed Sensor Data Fusion and Evaluation Architecture (MDSFEA) [23], as presented in Figure 2. The MDSFEA is an architecture suitable for data analysis ranging from homogeneous to heterogeneous datasets.

In Figure 2, data from thermal and radar SSs can be imported to the architecture with the help of the image and data import toolkits, respectively [47]. While the radar sensors data are stored in a CSV file, the thermal sensors data are stored as PNG files. Information such as the range of participants, speed, and the AAR from the radar SS were fused to the corresponding thermal images with the help of their timestamps using the data merging system. After the preliminary feature extraction that took place at the data merging system, Definitive Feature Extraction (DFE) began automatically. The DFE leveraged the data embedding toolkit to extract up to 1000 features from the datasets and represented them as vectors (n_0_ to n_999_) [48]. Although the MDSFEA description (in Figure 2) suits heterogeneous datasets such as those from thermal and radar SSs, it should be noted that the same architecture was used for the single and homogeneous datasets analysis.

Two main algorithms were used to further process the sensors datasets after the DFE stage namely, the HCA and the KMA. While the HCA used the Distance Toolbox (DT) to access the data embedder, the K-Means toolkit dissected the datasets (from the embedder) into clusters and conveyed them directly to the Test and Evaluation Toolkit (TET) (see Figure 2). The DT used Euclidean Distance Matrix (EDM) to further perform feature-based segregation on the datasets. The rationale for using the EDM includes the ability to perform distancing on raw data without previous analysis being affected by the addition of new data [49]. The HCA used Weighted Average Linkage (WAL) on the distanced features before the TET. The implementation of WAL on the datasets enhances feature prioritisation and the discriminant ability of the HCA algorithm [50,51,52]. DM models such as K-Nearest Neighbours (KNN), Support Vector Machine (SVM), Stochastic Gradient Descent (SGD), Random Forest (RF), and Neural Network (NN), amongst others, were used to evaluate the performance of the architecture.

**Figure 3:**
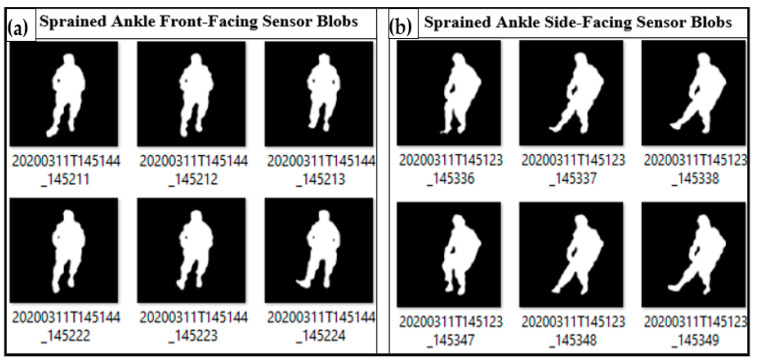
Instances of right-ankle and foot dorsiflexion, plantarflexion, eversion, and inversion as recorded by ITA-32 thermal sensors: (**a**) thermal blobs from the front-facing camera and (**b**) thermal blobs from the side-facing camera.

**Figure 4:**
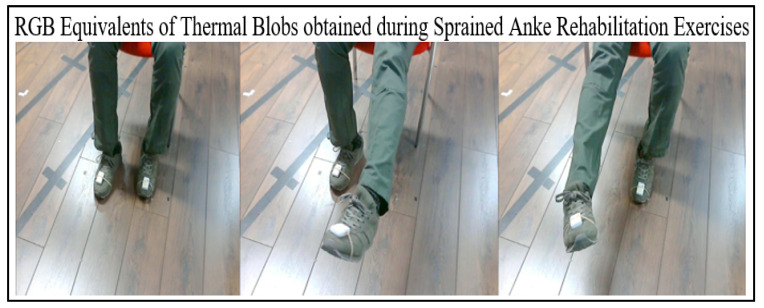
RGB equivalents of the thermal images recorded during sprained ankle rehabilitation exercise. A Shimmer-3 accelerometer is worn on metatarsals (on both legs) for ground-truth velocity measurements.

Thermal blobs from the ITA-32 thermal sensors were automatically binarised using a sequence of codes in MATLAB. To remove excess blobs from heating and electrical devices, a blob-based background subtraction algorithm was used [53,54,55]. Hence, the clear and distinct thermal blobs are presented in Figure 3a,b. The RGB equivalents demonstrating similar actions by the ITA thermal SS are shown in Figure 4.

## 4. Results

Data gleaned from the thermal SS indicated instances of the four directional movements on the right leg, as presented in Figure 3. To aid description, the last two digits of the thermal images’ timestamps are used for the analysis in this work. For example, thermal images code-named 20200311T145144_145211 and 20200311T145144_145222 (Figure 3a) are represented as T-11 and T-22, respectively.

**Figure 5:**
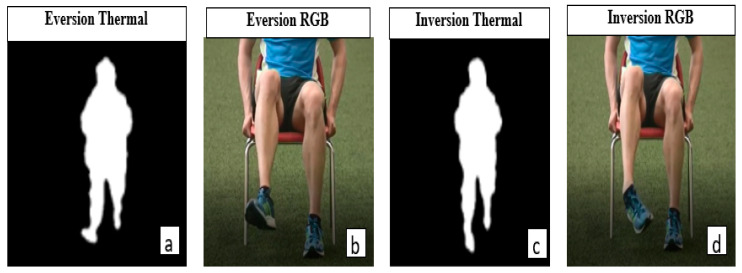
Instances of eversion and inversion of the ankle as presented by RGB and ITA-32 thermal sensors. (**a**) An instance of eversion by an ITA-32 thermal sensor, (**b**) an instance of eversion by an RGB camera, (**c**) an instance of inversion by an ITA-32 thermal sensor, and (**d**) an instance of inversion by an RGB camera.

In Figure 3, T-11 and T-49 indicated instances of eversion; T-12, T-22, and T-48 presented instances of inversion. Furthermore, plantarflexion is observed in T-37, T-38, and T-49, while dorsiflexion is indicated in T-23 and T-24. The background subtraction and image binarisation algorithms utilised in this study enhanced the granularity of the images, thus helping to clarify the direction of the ankle and foot by eliminating heat blobs from other individuals and devices [53,55]. A comparison of instances of eversion and inversion of the ankle as recorded by an RGB and the ITA (thermal) sensors are presented in Figure 5.

### 4.1. Single Dataset Analysis

On a single dataset analysis such as data from the front-facing ITA-32 thermal sensor, image embedding took place automatically after data import. This was followed by data distancing using the Inception-v3 Architecture (IV3A). The rationale for using IV3A included low computational requirements [56] and high performance in image analysis [57]. KMA and HCA were used simultaneously to perform CbyC on the datasets, and their results were evaluated using separate TETs. Moreover, while the K-Means toolbox was initialised with KMA to a maximum of 300 iterations, the hierarchical clustering toolkit used a 10-fold cross-validation function based on a 66% training set. The results of these analyses are presented in Table 2 and Table 3.

A close comparison of Table 2 and Table 3 indicated that the best average accuracies of the metrics were obtained using the HCA. Although KMA with four models—NN, SVM, SGD, and LR—obtained an accuracy of more than 98% for AUC, CA, F1, Precision, and Recall, the accuracies obtained in CN2 inducer and AdaBoost affected the overall accuracy of KMA. CN2 rule inducer had the least accuracy values for all the metrics in both KMA and HCA, as presented in Table 2 and Table 3.

### 4.2. Homogeneous Sensor Data Fusion

On homogeneous data fusion involving the front-facing and side-facing thermal sensors, data from the sensors were fused using a Matching Pairs of Rows Method (MPoRM). The rationale for using the MPoRM is that it allows for the proper fusion of homogenous data without information mismatch [47,58]. With the help of the image-embedding toolkit and its SqueezeNet architecture, a lightweight convolutional neural network model for image recognition [23], the merged data were routed to the Louvain Clustering Toolbox (LCT). The LCT automatically discovered eight clusters from the fused dataset by performing Euclidean distancing and Principal Component Analysis (PCA). The rationale for using EDM includes the ability to perform distancing on raw data without previous analysis being affected by the addition of new data [49]. On the other hand, using PCA helps to improve the clustering of the dataset. It differs from Linear Discriminant Analysis (LDA) because it is a variance-based algorithm, whereas LDA is based on class information [59,60]. Moreover, PCA is best suited for unsupervised data clustering, such as that used in this analysis [23].

The KMA involving up to eight clusters and 300 iterations was used for clustering the unified data. The rationale for using K-Means included simplicity and the ability to increase similarities within clusters and reduce the same outside the group [61,62]. This includes defining and associating k centroids for each cluster. Therefore, with the help of the KMA, the DM models related to the TET were capable of computing the accuracies of the processes based on a 10-fold cross-validation and average over classes, as presented in Table 4.

From Table 4, it can be observed that SGD and NN scored more than 98% in all the parameters such as AUC, CA, F1, Precision, and Recall. An accuracy of more than 90% was obtained in KNN, Decision Tree, SVM, SGD, RF, NN, and LR in all their parameters presented in Table 4. While SGD had the highest accuracy of more than 99% in all the parameters, the CN2 rule inducer scored the least. A further breakdown of the latter indicated the least accuracy of 85.6% for AUC and approximately 72% in CA, F1, Precision, and Recall. The average accuracies for all parameters were more than 90%.

### 4.3. Heterogeneous Sensor Data Fusion

Heterogeneous sensor data such as those from the side-facing ITA-32 thermal and radar sensors were also fused using the data merging toolkit. These data were first uploaded and processed using the import toolkits before being merged using the matching row appending rule. The fusion outcome was trained using the VGG-19, which is a-19-layer image recognition algorithm [63]. The merged data were normalised and distanced using the Manhattan Distance Metric (MDM). The rationale for using the MDM instead of others such as the cosine rule, included the grid-like behaviour of the former, which is a useful characteristic when dealing with heterogeneous data [64]. The accuracies of the CbyC with respect to heterogeneous data fusion are presented in Table 5.

As presented in Table 5, Stratified Cross-Validation (SCV) involved dividing the data into smaller sub-groups (strata). The rationale for using the SCV includes their ability to identify shared attributes in a dataset [64,65,66]. Moreover, all the metrics returned an average accuracy of more than 96%.

A further test on the averages of the models from Table 2, Table 3, Table 4 and Table 5 represented as KMA-A, HCA-A, SF-Fusion, and Rad-T Fusion, respectively, is presented in Table 6. The models considered were KNN, Decision Tree, SVM, SGD, RF, NN, Naives Bayes, and AdaBoost. However, models that were not common to the tested models in a column were excluded from the test. The rationale for excluding them was to achieve a balanced and unbiased dataset at each instance. Considering the averages presented in Table 6, the highest average accuracy value was obtained in Rad-T Fusion as 96.9%.

A two-sample T-Test of the KMA-A values and the HCA-A values at 95% confidence interval indicated that there was no significant difference (*p* = 0.448) between the samples. This implied that the accuracy values obtained in Table 2 and Table 3 were within a close range. On the other hand, a two-sample T-Test of the KMA-A and the Rad-T Fusion values (Table 6) indicated a significant difference between the values (*p* = 0.032). Similarly, a two-sample T-Test between SF-Fusion and Rad-T values also showed a significant difference between the values (*p* = 0.034). The significant differences (*p* = 0.032 and *p* = 0.034) obtained in the two instances involving Rad-T indicated that the accuracy values of the heterogeneous sensor fusion datasets (in Table 5) were distinct and different from those obtained from the homogenous and single datasets. Hence, a descriptive analysis of the parameters in Table 6 (KMA-A, HCA-A, SF-Fusion, and Rad-T Fusion) based on one-way ANOVA is presented in Table 7. The row containing Naive Bayes was not included in the computation of the mean because of the non-availability of a value for HCA-A.

In Table 7, one-way ANOVA involving KMA-A, HCA-A, SF-Fusion, and Rad-T Fusion indicated the latter (Rad-T Fusion) as the parameter with the highest range of values at 95% confidence interval. From the result, Rad-T Fusion still maintained its highest average value. It also has the least standard deviation of 3.9, which further indicated the proximity of its values.

## 5. Discussion

### 5.1. Discussion on Research Findings

The average results in Table 6 and Table 7 indicated that the highest average percentage accuracy was obtained in the heterogeneous datasets involving thermal and radar SS as 96.9%. Hence, it can be suggested that complementary monitoring involving heterogeneous SSs, such as the approach demonstrated in this study, yields higher accuracy compared with a single or homogeneous monitoring solution.

SPAREs using USSs such as the MC-FMCW-M radar and ITA-32 thermal SSs offers the ability to visualise the movement of the ankle in the four fundamental movements of the human ankle. The level of details such as the direct computation of the speed and range of motion of the ankle presents the FMCW radar variants as better alternatives to wearables such as the S3BAs, which are not capable of direct computation of speed. In addition, the privacy-friendly images obtained from the ITA-32 thermal SS are best suited for home-based monitoring compared with the RGB images produced by video cameras, which can negatively affect their users’ privacy.

### 5.2. Comparison of the Present Work with Related Work

Data gleaned from the USSs in this work required less storage space, less computational time, and fewer resources compared with the RGB-based approach proposed in [36]. Additionally, our approach is privacy-friendly compared with the work in [38]. Compared to similar research in [67,68], our work utilised a cluster-based approach; it computes the accuracy of the clustered datasets and compares results from single, homogeneous, and heterogeneous datasets using statistical methods such as T-Test and ANOVA. Although the use of wearables in SPAREs proposed in [7,26] has been shown to provide high usability and accuracy, our approach featured additional information such as participants’ postures, which can help therapists determine if SPAREs have been performed as prescribed. Additionally, frequent (re)charging processes, which can result in data loss or disruption, were not required in our work, since the sensors were plugged in to AC supply. Hence, the proposed USSs are better candidates for home-based monitoring of SPAREs compared with wearables such as the battery-powered gyro-based systems.

Whilst some research [8,29,69] in SPAREs has been through human observation without a clear indication of the monitoring accuracy, the present work indicated the accuracy of the monitoring processes. Another advantage of this work is the use of heterogeneous USSs compared with wearables proposed in [7,26,70], which could be a source of discomfort to the technology users.

The MDSFEA proposed in the present work offered added advantages in feature extraction, visualisation, and model evaluation compared with the widely used method in [26,71]. Instead of processing each model separately, which can be time consuming, the models all learnt from the TETs and simultaneously produced comprehensive results on the TETs. The fusion of data from the SSs gives useful and additional information such as the speed and the AAR of the ankle at every second during the exercise. Information such as the posture, speed, range, and the AAR related to the ankle movement during SPARE can help physiotherapists ascertain if exercises have been performed as prescribed.

### 5.3. Limitations of the Present Work

One of the limitations of this study is that homogeneous data fusion involving the front-facing and the side-facing ITA-32 thermal sensors did not produce evaluation results with HCA when the datasets were fused with MPoRM. This issue was resolved by using the “instance IDs” of the datasets for results scoring and evaluation. Another limitation is that ML models such as LR and Naive Bayes were not computed for all dataset types. The rationale for this exclusion included their incompatibility with the datasets and the data fusion algorithms.

## 6. Conclusions

This paper presented the use of privacy-friendly USSs such as thermal and radar sensors to monitor SPAREs in home environments. Data gleaned from the USSs were analysed and fused using the MDSFEA on instances involving single, homogeneous, and heterogeneous SS. Experimental results from model averages indicated mean accuracy values of 91.9%, 92.4%, 92.0%, and 96.9% for KMA-A, HCA-A, SF-Fusion, and Rad-T Fusion, respectively, with models such as KNN, Decision Tree, SVM, SGD, RF, NN, Naïve Bayes, and CN2 inducer. Descriptive analysis of the model averages further indicated that the highest average percentage accuracy was obtained in the heterogeneous datasets involving thermal and radar sensors, demonstrating the added advantages of using heterogeneous USSs and data fusion in home-based SPAREs.

## Figures and Tables

**Table 1:** Comparison of Sensing Solutions (SSs) in Sprained Ankle Rehabilitation Exercises (SPAREs) monitoring.

Sensing Solution	Parameters	Home Environment	Advantages	Disadvantages	Example Studies in SPAREs
Wearables, e.g., accelerometer, gyroscope, etc.	Angular acceleration and range of motion.	Yes	Excellent sensitivity, privacy-protected data, inexpensive, small, and light weight.	Wearability, battery-life issues, forgetfulness to wear, data disruptions.	Gyro-based system and WiFi-based monitoring [7].
RGB, e.g., Vicon Cameras.	Speed, range of motion, frequency, angles of motion.	Yes	High frame-rate data acquisition and high-quality images.	High storage space requirement, expensive, privacy issues.	Video and game-based approach in SPAREs [36].
Depth, e.g., Kinect.	Posture estimation, speed, range of motion, joint angles of motion.	Yes	Joints and 3D features reconstruction, inexpensive.	Reflection problems, short-range, environment interference.	Joint angles and posture acquisition [16,31,32].
Thermal, e.g., Infrared Thermopile Array.	Posture and joint angles estimation, anatomical information.	Yes	Privacy protected images, illumination independent, inexpensive.	Slow response time, low sample rate.	Privacy-friendly SPAREs monitoring [39].
Radar, e.g., FMCW Radar.	Speed, range of motion, frequency, angles of motion.	Yes	Privacy-protected data, illumination independence, no interference with legacy systems, inexpensive.	No images for low-cost sensors.	Home-based ankle rehabilitation [6].

**Table 2:** Evaluation results showing the accuracies of data mining models during Classification-by-Clustering of a set of sprained ankle rehabilitation exercises data using K-Means++ Algorithm (KMA).

Stratified 10-Fold Cross-Validation of Side-Facing ITA-32 Sensor Data with K-Means++					
Model	AUC (%)	CA (%)	F1 (%)	Precision (%)	Recall (%)
KNN	98.2	93.0	93.0	93.1	93.0
Decision Tree	91.4	90.1	90.1	90.1	90.1
SVM	99.9	98.2	98.2	98.2	98.2
SGD	99.5	99.3	99.3	99.3	99.3
RF	98.0	89.5	89.5	89.5	89.5
NN	99.7	98.6	98.6	98.6	98.6
Naïve Bayes	92.9	80.5	80.7	81.4	80.5
LR	99.9	98.9	98.9	98.9	98.9
CN2 Rule Inducer	85.6	71.8	71.9	72.3	71.8
AdaBoost	90.5	87.3	87.3	87.3	87.3
Average	95.6	90.7	90.7	90.9	90.7

Legend: KNN = K-Nearest Neighbours, LR = Logistic Regression, NN = Neural Network, RF = Random Forest, SGD = Stochastic Gradient Descent, SVM = Support Vector Machine, CA = Classification Accuracy, and AUC = Area under the Curve.

**Table 3:** Evaluation results showing the accuracies of data mining models during Classification-by-Clustering of a set of sprained ankle rehabilitation exercises data using the Hierarchical Clustering Algorithm (HCA).

Stratified 10-Fold Cross-Validation of Side-Facing ITA-32 Sensor Data with HCA					
Model	AUC (%)	CA (%)	F1 (%)	Precision (%)	Recall (%)
KNN	99.5	95.6	95.6	95.7	95.6
Decision Tree	90.6	89.7	89.7	89.7	89.7
SVM	99.6	95.1	95.2	95.5	95.1
SGD	99.2	98.9	98.9	98.9	98.9
RF	99.3	94.9	94.9	94.9	94.9
NN	99.4	98.1	98.1	98.1	98.1
LR	100	99.6	99.6	99.6	99.6
CN2 Rule Inducer	84.8	71.4	71.4	71.4	71.4
AdaBoost	91.9	89.2	89.2	89.2	89.2
Average	96.0	92.5	92.5	92.6	92.5

Legend: KNN = K-Nearest Neighbours, LR = Logistic Regression, NN = Neural Network, RF = Random Forest, SGD = Stochastic Gradient Descent, SVM = Support Vector Machine, CA = Classification Accuracy, and AUC = Area under the Curve.

**Table 4:** Evaluation results of the front and side-facing ITA-32 thermal sensor data using a 10-fold cross-validation and the average of classes. The fused data were obtained during sprained ankle rehabilitation exercises.

Data Fusion of Side-Facing and Front-Facing ITA-32 Sensors (SF-Fusion)					
Model	AUC (%)	CA (%)	F1 (%)	Precision (%)	Recall (%)
KNN	98.2	93.0	93.0	93.1	93.0
Decision Tree	91.4	90.1	90.1	90.1	90.1
SVM	99.9	98.2	98.2	98.2	98.2
SGD	99.3	99.1	99.1	99.1	99.1
RF	98.0	91.5	91.5	91.5	89.5
NN	99.7	98.6	98.6	98.6	98.6
Naïve Bayes	92.9	80.5	80.7	81.4	80.5
LR	99.9	98.9	98.9	98.9	98.9
CN2 Rule Inducer	85.6	71.8	71.9	72.3	71.8
AdaBoost	90.4	87.3	87.3	87.3	87.3
Average	95.5	90.9	90.9	91.1	90.7

Legend: KNN = K-Nearest Neighbours, LR = Logistic Regression, NN = Neural Network, RF = Random Forest, SGD = Stochastic Gradient Descent, SVM = Support Vector Machine, CA = Classification Accuracy, and AUC = Area under the Curve.

**Table 5:** Evaluation results of ITA-32 thermal and FMCW radar sensor data using a stratified 10-fold cross-validation and the average of classes. The fused data were obtained during sprained ankle rehabilitation exercises.

Data Fusion of ITA-32 Thermal and Radar Sensors (Rad-T Fusion)					
Model	AUC (%)	CA (%)	F1 (%)	Precision (%)	Recall (%)
KNN	99.5	99.3	99.3	99.3	99.3
Decision Tree	99.7	99.5	99.5	99.5	99.5
SVM	99.6	95.1	95.1	95.5	95.1
SGD	99.1	98.8	98.8	98.8	98.8
RF	99.0	94.4	94.4	94.4	94.4
NN	99.7	97.4	97.4	97.4	97.4
Naïve Bayes	98.9	95.6	95.7	95.8	95.6
CN2 Rule Inducer	99.6	99.5	99.5	99.5	99.5
AdaBoost	90.8	87.8	87.8	87.8	87.8
Average	98.4	96.4	96.4	96.4	96.4

Legend: KNN = K-Nearest Neighbours, NN = Neural Network, RF = Random Forest, SGD = Stochastic Gradient Descent, SVM = Support Vector Machine, CA = Classification Accuracy, and AUC = Area under the Curve.

**Table 6:** Model averages from KMA (Table 2), HCA (Table 3), SF-Fusion (Table 4), and Rad-T (Table 5).

Model	KMA-A (%)	HCA-A (%)	SF-Fusion (%)	Rad-T Fusion (%)
KNN	94.1	96.4	94.1	99.3
Tree	90.1	89.9	90.4	99.5
SVM	98.5	96.1	98.5	96.1
SGD	99.3	99.0	99.1	98.9
RF	91.2	95.8	92.4	95.3
NN	98.8	98.4	98.8	97.9
Naive Bayes	83.2	NA	83.2	96.3
CN2 Rule Inducer	74.7	74.1	74.7	99.5
AdaBoost	87.9	89.7	87.9	88.4
Average	91.9	92.4	92.0	96.9

KNN = K-Nearest Neighbours, NN = Neural Network, RF = Random Forest, SGD = Stochastic Gradient Descent, SVM = Support Vector Machine, CA = Classification Accuracy, and AUC = Area under the Curve. NA = Not available.

**Table 7:** Descriptive analysis of KMA-A, HCA-A, SF-Fusion, and Rad-T Fusion average values.

Descriptive Analysis of the Parameters				
Parameters	N	Mean	StDev	95% CI
KMA-A	8	91.9	8.2	(86.6, 97.2)
HCA-A	8	92.4	8.2	(87.1, 97.7)
SF-Fusion	8	92.0	8.1	(86.7, 97.3)
Rad-T Fusion	8	96.9	3.9	(91.6, 100.0)

Legend: N = total number of rows used for the analysis, CI = Confidence Interval, StDev = Standard Deviation.

## Data Availability

Not Applicable.
